# Enhancement of the Optical and Dielectric Properties at Low Frequency of (Sr_1−x_Ca_x_)_5_Ti_4_O_13_, (0 ≤ x ≤ 0.06) Structure Ceramics

**DOI:** 10.3390/mi13111824

**Published:** 2022-10-26

**Authors:** Sara J. Ahmed, Asad Ali, Abid Zaman, Aiyeshah Alhodaib, Abdulaziz H. Alghtani, Imen Bejaoui, Vineet Tirth, Ali Algahtani, Mohsin Khan, Abdullah, Mohammed Aljohani

**Affiliations:** 1 Medical Physics Department, Al-Mustaqbal University College, Babylon 51001, Iraq; 2 Department of Physics, Government Postgraduate College, Nowshera 24100, Pakistan; 3 Department of Physics, Riphah International University, Islamabad 44000, Pakistan; 4 Department of Physics, College of Science, Qassim University, Buraydah 51452, Saudi Arabia; 5 Department of Mechanical Engineering, College of Engineering, Taif University, P.O. Box 11099, Taif 21944, Saudi Arabia; 6 Department of Chemistry, College of Arts and Science Sarat Abidah, King Khalid University, Abha P.O. Box 9004, Saudi Arabia; 7 Mechanical Engineering Department, College of Engineering, King Khalid University, Abha 61421, Saudi Arabia; 8 Research Center for Advanced Materials Science (RCAMS), King Khalid University Guraiger, P.O. Box 9004, Abha 61413, Saudi Arabia; 9 Department of Physics, Government Post Graduate College, Karak 27200, Pakistan; 10 Department of Chemistry, College of Science, Taif University, Taif 21944, Saudi Arabia

**Keywords:** (Sr_1−x_Ca_x_)_5_Ti_4_O_13_ ceramics, XRD, FT-IR optical, electric properties

## Abstract

Low loss Ruddlesden–Popper (RP) series, i.e., (Sr_1−x_Ca_x_)_5_Ti_4_O_13_, 0.0 ≤ x ≤ 0.06, has been synthesized by a mixed oxide route. In this work, the substitution of Ca^2+^ cation in Sr_5_Ti_4_O_13_ sintered ceramics was chosen to enhance the structural, optical, and dielectric properties of the product. It was found that the Ca^2+^ content has significant effects on enhancing the dielectric properties as compared to Mn and glass additions. It was observed that the relative density, band gap energy, and dielectric loss (tangent loss) increase while relative permittivity decreases along with Ca^2+^ content. High relative density (96.7%), low porosity, and high band gap energy (2.241 eV) values were obtained in (Sr_1−x_Ca_x_)_5_Ti_4_O_13_, 0.0 ≤ x ≤ 0.06 sintered ceramics. These results will play a key role in the application of dielectric resonators.

## 1. Introduction

Recently, the Sr based Ti family has come to play a key role in the development of wireless communication technologies [1]. The low loss dielectric ceramics with good dielectric properties and temperature stability are widely used in the applications of dielectric resonator antennas [2,3,4,5]. To overcome the miniturized devices requirements, the ceramic dielectrics must have maximum values of relative permittivity (εr) and low dielectric losses [6,7,8,9]. Besides the poor sintering temperature, the barium magnescium tanatalate material have optimum dielectric properties, which is used further in the applications of dielectric resonator devices [10,11,12]. On the other side, compound, i.e., CaTiO_3_-MgTiO_3_, ceramics posses good dielectric properties, i.e., (ε_r_ = 21.2, Q × *f* = 56,200 GHz, τ*_f_* = ±0 ppm/°C). Lately, the RP series ceramics have been the subject of numerous scientific studies, with results showing their interesting dielectric properties [13,14,15,16]. A_n+1_B_n_O_3n+1_ is the general formula of RP-series, and the phase analysis consists of n—blocks of octahedra (BO6) corner-sharing which construct a layered peroveskite like structure. In previous research, it has been reported that the compound, i.e., SrLn_2_Al_2_O_7_ and MLnAlO_4_ (Ln = Nd, La, & Sm, M = Ca, Sr), ceramics along with n = 1 and n = 2 show good dielectric properties. The RP series posses bulk structure and optimum dielectric properties, i.e., ε_r_ = 16.0 to 19.0, Q × *f* = 54,600–69,500 GHz and τ*_f_* = −1–−32 ppm/°C for n = 1; ε_r_ = 17.9 to 21.6, Q × *f* = 64,680–71,680 GHz & τ*_f_* = +4–−22.1 ppm/°C for n = 2, have been reported [13,14,15,16,17]. Fan Yi et al. reported the modification in phase, microstructure, and dielectric properties of RP-series [14,15]. Actually, this impression has been realized in M^2+^/Ti^4+^ cation substitution in MLnAlO_4_ calcined ceramics, which further modified the phase, microstructure, optical, and dielectric properties, i.e., (ε_r_ = 18.2 to 21.4, Q × *f* = 75,000 GHz to 96,500 GHz and τ*f* ~ ±0 ppm/°C) [18,19,20]. On the other hand, the dielectric properties, especially quality factor value, are not too good. In RP-series, at n = 2, the ceramic compound, i.e., SrLn_2_Al_2_O_7_ generally has low dielectric losses and good relative permittivity values as compared to MLnAlO_4_ ceramics. Moreover, many researchers have reported the optimum dielectric properties among the RP series [13,14,15,16,17,18,19,20,21]. The RP-series, such as Sr_2_La(A_3_ + B_4_)O_7_ (A = Fe, Cr, B = Mn, Ti), has been investigated already in a literature review, and a new RP compound, (Sr_2_LaAlTiO_7_), has been synthesized and analyzed as a new low loss ceramic material [22,23,24,25,26].

In the present work, the good results on structural, optical, and dielectric properties (at low frequency) of Sr_5_Ti_4_O_13_ based structure ceramics will be studied. These results will be modified by making some doping elements at A-site cation in the base product.

## 2. Experimental Procedure

The solid solution of (Sr_1−x_Ca_x_)_5_Ti_4_O_13_, 0 ≤ x ≤ 0.06 ceramic was processed by using high grade pure carbonates and oxide powders, i.e., SrCO_3_ (99.95%), CaCO_3_ (99.9%), and TiO_2_ (99.5%) as raw materials. The resultant stoichiometric ratio of the raw materials were mixed properly and then milled using horizontal ball milling with zirconia media in distilled water for 24 h and then calcined for 3 h in air at 980 °C. After re-milling, the calcined powder was mixed along with polyvinyl alcohol (PVA) solution at 4 wt.% and then made into green pellets of 2–3 mm thickness and 10–12 mm diameter. Then, the green pellets were kept in high energy furnace at 1200 °C sintering temperature for 3 h in air to dense the pellets. After sintering, the pellets were cooled to 600 °C at the rate of 10 °C/min and then cooled to room temperature inside the furnace further. The bulk density was calculated by using the Archimedes principle method for all the pellets. The phase analysis was identified by using X-ray diffraction (XRD, RIGAKU D/max 2550/PC, Rigaku Co-Tokyo Japan) with CuKα radiation. The surface morphology of the thermally etched and gold coated samples was studied using scanning electron microscopy (SEM, S3400; Hitachi, Tokyo, Japan). The relative permittivity (ε_r_) and tangent loss were measured by the parallel plate capacitor method using vector-network Analyzer (E8363B, Agilent Technologies Inc., Santa Clara, CA, USA) [27]. At least four samples have been analyzed to ensure the accuracy of data. The reciprocal of Q-factor is the tangent loss (tanδ = 1/Q) [28].

## 3. Results and Discussion

### 3.1. Phase Analysis

**Figure 1:**
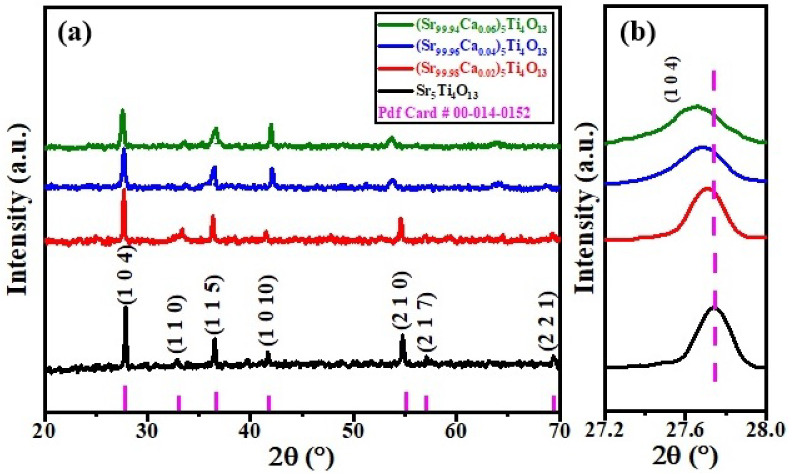
(**a**) XRD pattern of (Sr_1−x_Ca_x_)_5_Ti_4_O_13_, 0 ≤ x ≤ 0.06 sintered ceramics (**b**) zoomed view of peak (1 0 4) shifted toward the lowest angle.

**Figure 2:**
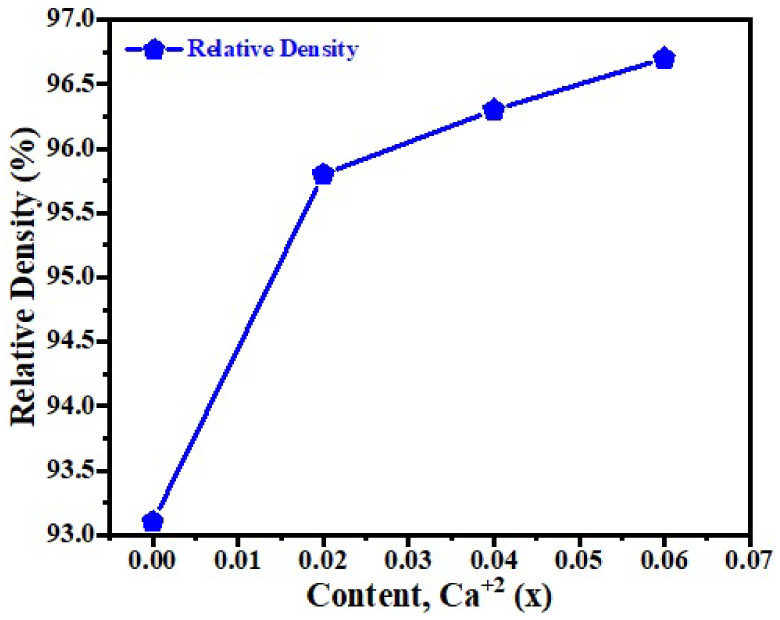
The relative density of (Sr_1−x_Ca_x_)_5_Ti_4_O_13_, 0 ≤ x ≤ 0.06 ceramics.

Figure 1 shows the XRD patterns of RP series of (Sr_n+1_Ti_n_O_3n+1_) sintered ceramic for n = 4. The patterns revealed the tetragonal structure of RP series along with space-group (I4/mmm) matched to PDF card number 89-1383. The structure of the phase (at n = 4) was attained by put in a rock-salt type Sr-O layers, the strontium based titanates along with direction [001], resulting consecutive perovskite pieces due to shifting by direction 1 ÷ 2 [111], w.r.t the unit cell of RP series. The known RP structure has closely alike lattice parameters i.e., (a = b = 0.385 to 0.389 nm) but c = 2.812 nm for n = 4 [27,28,29,30,31,32]. The variation of lattice parameters and volume of the synthesized samples with Ca^2+^ contents as shown in Table 1. The shifting of peaks to lowest Bragg’s angles were due to the difference of ionic radii of Sr^2+^ and Ca^2+^ cations as shown in Figure 1b. No secondary phase has been observed and revealed the single phase of Sr_n+1_Ti_n_O_3n+1_ (n = 4) sintered ceramic. Figure 2 shows the variation of relative density with Ca^2+^ contents of Sr_n+1_Ti_n_O_3n+1_ (n = 4) sintered ceramics. It has been noted that the relative density increases with the Ca^2+^ content, which further modified the optical and dielectric properties. The highest values of relative density is (96.7%) of Sr_n+1_Ti_n_O_3n+1_ (n = 4) sintered ceramics was observed at x = 0.06 content.

### 3.2. Surface Morphology

**Figure 3:**
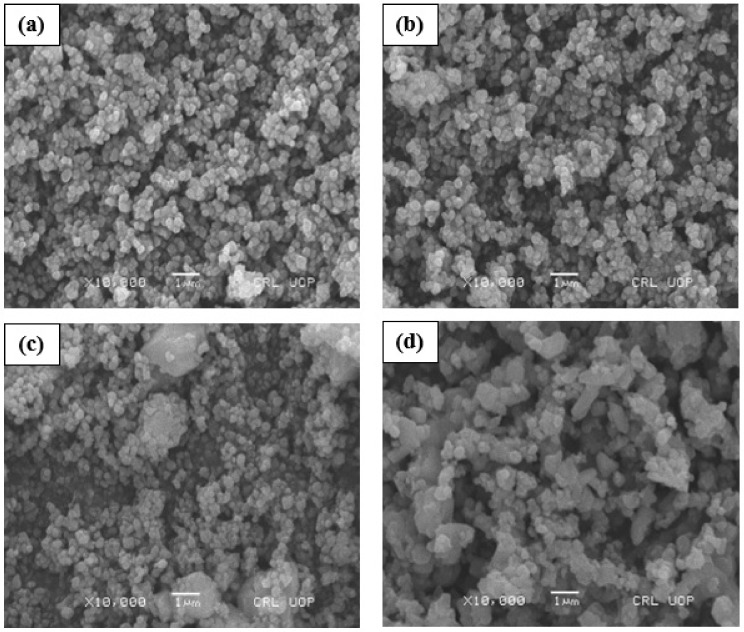
SEM images of polished & thermally etched samples of (Sr_1−x_Ca_x_)_5_Ti_4_O_13_, 0 ≤ x ≤ 0.06 sintered ceramics (**a**) x = 0.00, (**b**) x = 0.02, (**c**) x = 0.04 and (**d**) x = 0.06.

Figure 3 shows the SEM images of the gold coated samples of (Sr_1−x_Ca_x_)_5_Ti_4_O_13_, 0.00 ≤ x ≤ 0.06 sintered ceramics. The variation of relative densities and grain size of all the samples has been investigated. The SEM micrographs of (Sr_5_Ti_4_O_13_) green pellets with doping of Mn or glasses at different sintering temperature were studied by many scientific researchers [26]. It has been reported that the base product have small crystallite size and less porosity, which may be affected by the surface strain. However, new grains and porosity were produced by adding some dopant elements in (Sr_5_Ti_4_O_13_) sintered ceramic [27,28,29,30]. Ca^2+^ concentration has been observed to increase the porosity and grain size of all samples in (Sr_5_Ti_4_O_13_) sintered ceramic. These factors will affect the structure, optical, and dielectric properties of the base product. In order to improve these properties, numerous studies have examined the synthesis settings used to create various dopants in the base product [31,32].

### 3.3. Fourier Transform Infra-Red (FTIR) Spectroscopy

**Figure 4:**
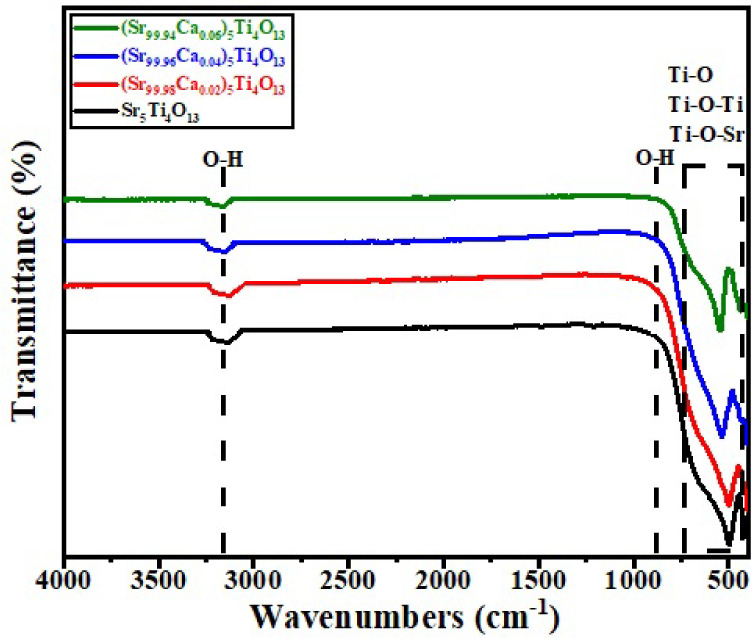
FTIR spectra of (Sr_1−x_Ca_x_)_5_Ti_4_O_13_, 0 ≤ x ≤ 0.06 sintered ceramics.

Figure 4 shows the FTIR spectra of (Sr_1-x_Ca_x_)_5_Ti_4_O_13_, 0 ≤ x ≤ 0.06 sintered ceramic. FTIR spectrometer plays a key role to characterize the vibrational stretching and un-stretching mode of the base sample synthesized by chemical reaction route [33,34]. The vibrational stretching mode (O-H) was observed with variable wave number (K = 2π/λ) i.e., 900.0 cm^−1^, and 3200.0 cm^−1^. This mode of vibration is produced by the absorption of vapors during synthesis process. Only asymmetric mode at wave number (3700.0 per cm) was recorded in the base product which shown carboxylates family [35]. In this characterization the normal stretching mode was observed at wave number (500.0 per cm).

### 3.4. UV Spectroscopy

**Figure 5:**
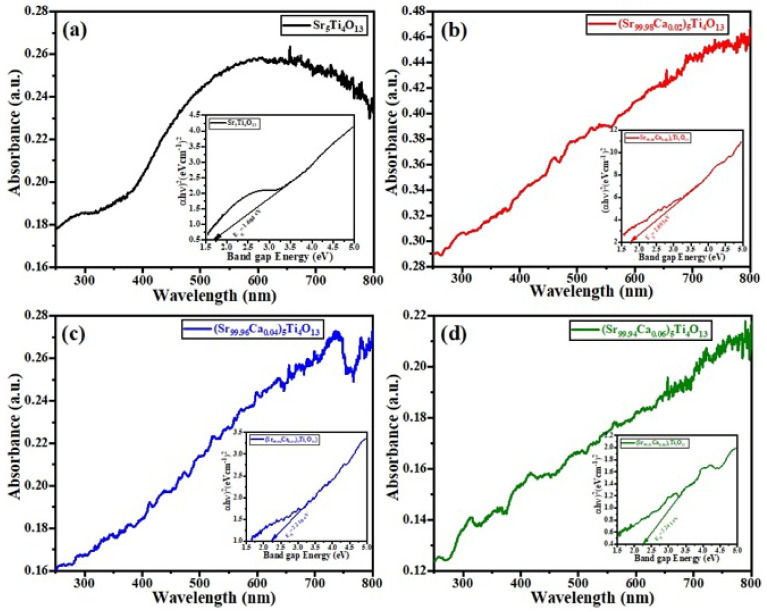
Band gap energies of (Sr_1−x_Ca_x_)_5_Ti_4_O_13_, 0.0 ≤ x ≤ 0.06 ceramics i.e., (**a**) 1.684 eV (**b**) 2.093 eV (**c**) 2.216 eV & (**d**) 2.241 eV.

Figure 5 shows the UV-spectra of (Sr_1−x_Ca_x_)_5_Ti_4_O_13_, 0.00 ≤ x ≤ 0.06 sintered ceramics. Many of the researchers reported that the Sr_5_Ti_4_O_13_ base sample was found to be transparent for white light [36]. It is very important to note that the compound, i.e., Sr_5_Ti_4_O_13_, is translucent for visible light. The band structure and electronic transition were characterized using photon energy [37]. The electron needs to execute the inner shell transition in order to obtain the optical bandgap energy. This optical bandgap energy strongly depends upon the coefficient of absorption (*α*), which was calculated using Equation (1) [25].

(1) $$\alpha =\frac{A{\left(hv-E_{g}\right)}^{1/2}}{hv}$$

where *E_g_* = bandgap energy, *A* = constant of proportionality, and *hv* = photon energy.

The coefficient of absorption will be defined how distant light of specific wavelength can be penetrated into material before being absorbed. When light absorbed poorly by material have low coefficient of absorption looks to be thin at specific wavelength. The unit of absorption coefficient is (cm^−1^). The band gap energy of all the samples was calculated using the Tauc plots method. It was reported that the values of band gap energy increase with Ca^2+^ content.

### 3.5. Photoluminescence (PL) Spectroscopy

**Figure 6:**
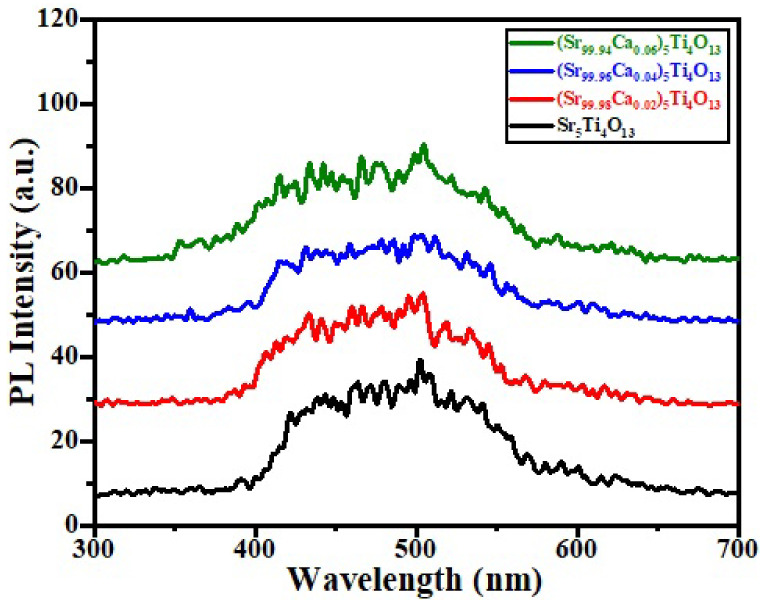
PL spectra of (Sr_1−x_Ca_x_)_5_Ti_4_O_13_, 0.0 ≤ x ≤ 0.06 ceramics.

Figure 6 shows the photoluminescence (PL) spectroscopy of (Sr_1−x_Ca_x_)_5_Ti_4_O_13_, 0.00 ≤ x ≤ 0.06 sintered ceramics. The emission line spectrum will be produced by the recombination of holes and electron charge carriers. Using the equation (E = hc/λ), where E = optical excitation energy, h = Plank’s constant (~6.63 × 10^−34^ Js) c = speed of light (3 × 10^8^ m/s) and λ is the emission wavelength, we can find the value of excitation energy of all the samples.

Emission at photoluminescence peak of the samples has been noted at the range of ~400–550 nm. Multiple photonic processes such as PL have certain common uses, and PL is a multiple photonic process that has some typical applications, i.e., (i) determination of band gape energy, (ii) material quality, as well as (iii) molecular structure and crystallinity, reported by many researchers [38,39]. It has been observed that the broader emission spectra were located near to ~2.48 eV (excitation energy) and wavelength (~500 nm) which is larger than bandgap energy of all the samples may be occurs due to the presence of impurities. In the photoluminescence spectrum, the cyan color may occur due to the oxygen vacancy [40].

### 3.6. Low Frequency Dielectric Properties

**Figure 7:**
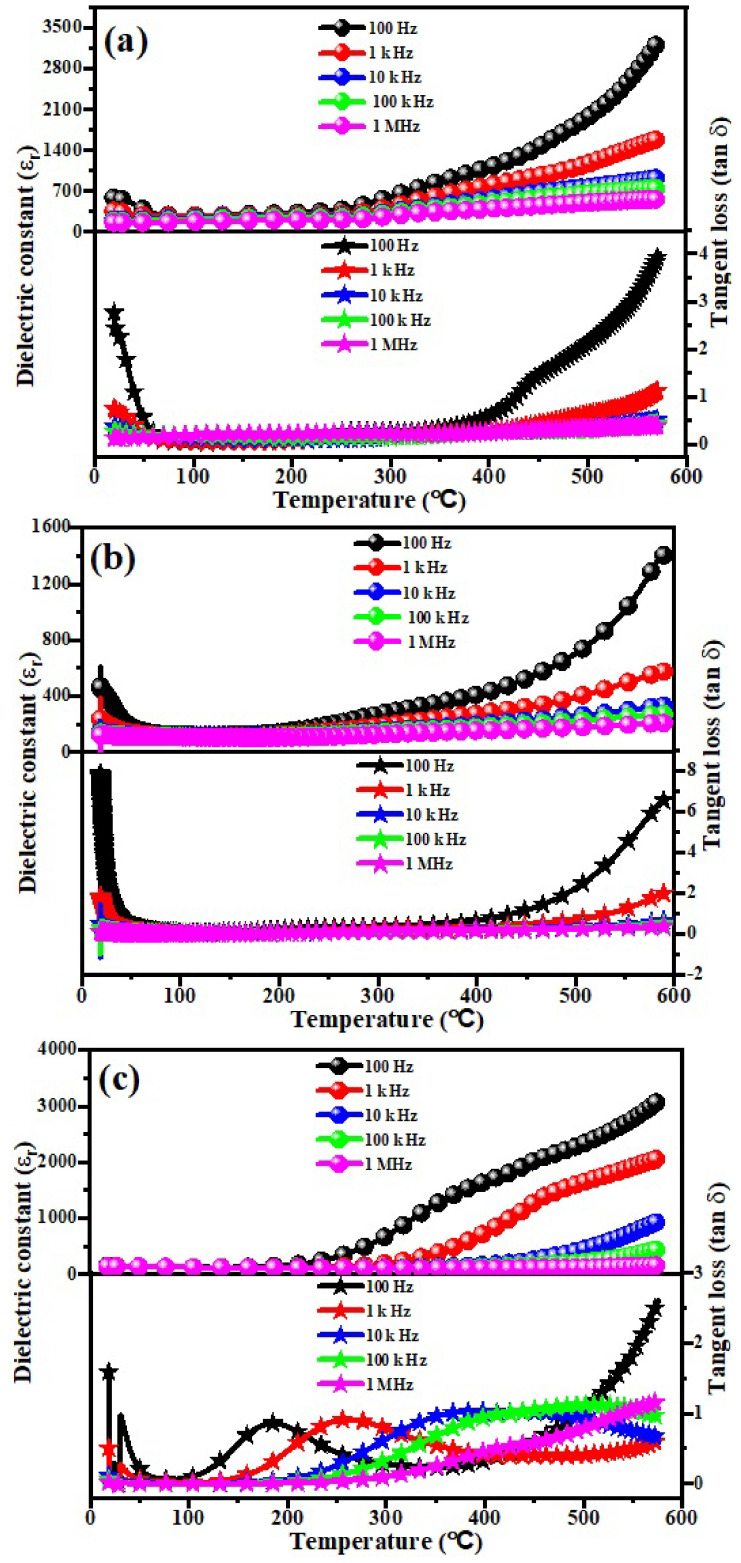

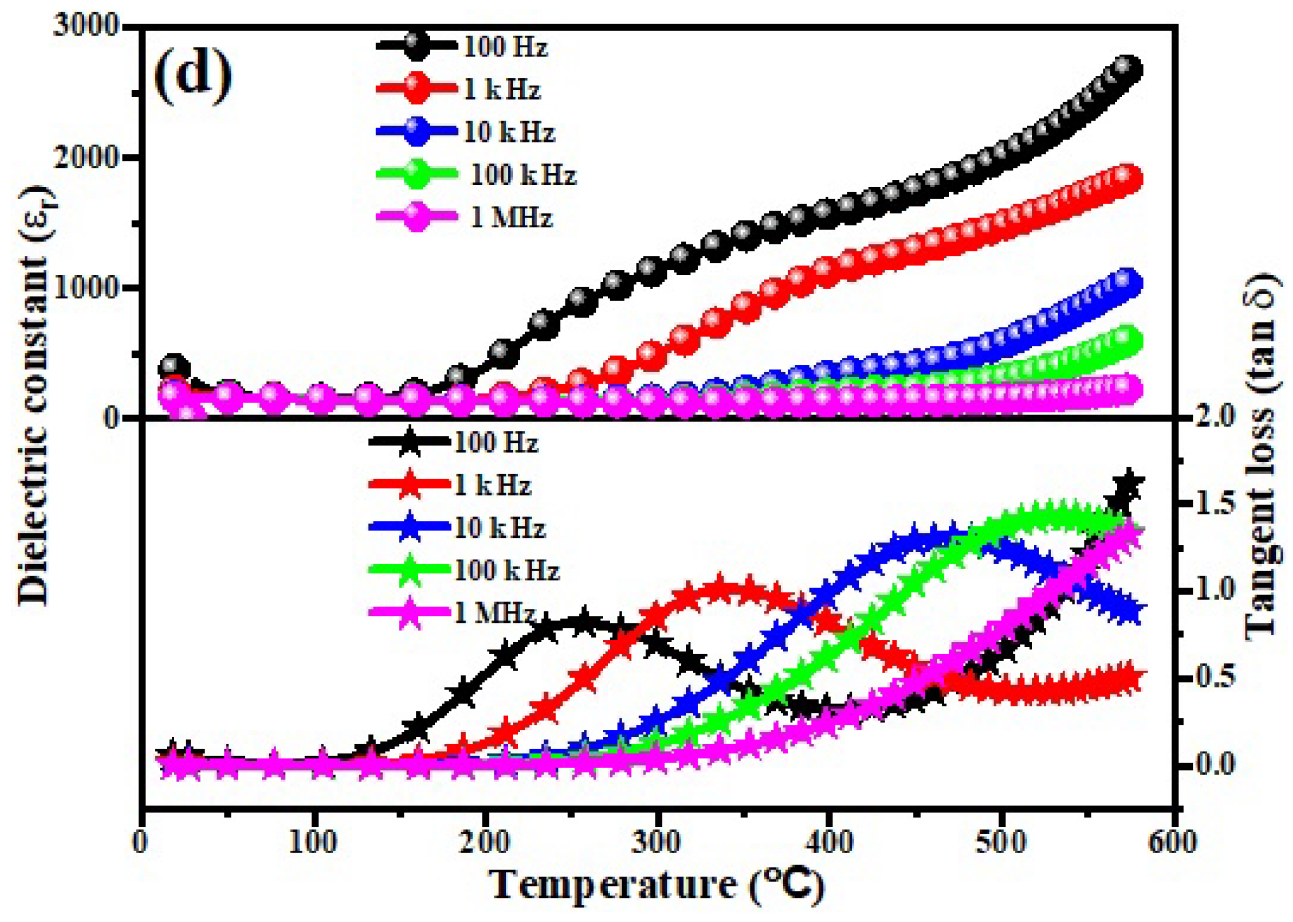
Variation of εr and tanδ with temperature for the (Sr_1−x_Ca_x_)_5_Ti_4_O_13_, 0.0 ≤ x ≤ 0.06 sintered ceramics i.e., (**a**) x = 0.00, (**b**) x = 0.02, (**c**) x = 0.04 & (**d**) x = 0.06.

The low frequency dielectric properties of all the synthesized samples sintered at 1200 °C for 3 h in air were better due to their high relative densities. The variation of relative permittivity (εr) and tangent loss (tanδ) with varying temperature was measured at 100 Hz–1 MHz for (Sr_1−x_Ca_x_)_5_Ti_4_O_13_, 0.0 ≤ x ≤ 0.06 sintered ceramics using the vector network analyzer, as shown in Figure 7. Strong irregularity in relative permittivity (ε_r_) and tangent loss (tanδ) were observed for the contents (at x = 0.0 and 0.02), which shows the transition of ferroelectric to Para electric phases. The same behavior was recorded in the values of ‘ε_r_’ and ‘tanδ’ for Ba_5-x_Sr_x_DyTi_3_V_7_O_30_ (0 ≤ x ≤ 5) sintered ceramics at temperatures of 430 °C, 350 °C, 325 °C, 85 °C, and 42 °C, respectively [41,42]. The lowest value of ε_r_ (~1400) was observed for (Sr_1−x_Ca_x_)_5_Ti_4_O_13_, (composition with x = 0.02) at 100 Hz frequency, and found to decrease with increasing operating frequency, which may be due to the interfacial polarizations. Moreover, the value of *ε*r decreased with increasing Ca^2+^ contents, which is due to the difference of ionic polarizebilities of Ca^2+^ (3.16 Å^3^) and Sr^2+^ (4.24 Å^3^) [43,44,45]. It has been revealed that the value of tangent loss increases with temperature due to the proces of conductivity and different types of polarizations at low frequency [21]. The lower value of the tangent loss was reported at 1 MHz operating frequency for the base sample. The variations in both the quantities may be due to the difference in the values of dielectric polarizabilities [46]. Generally, tanδ decreases when high cation ions are replaced by smaller cation ions [47].

**Figure 8:**
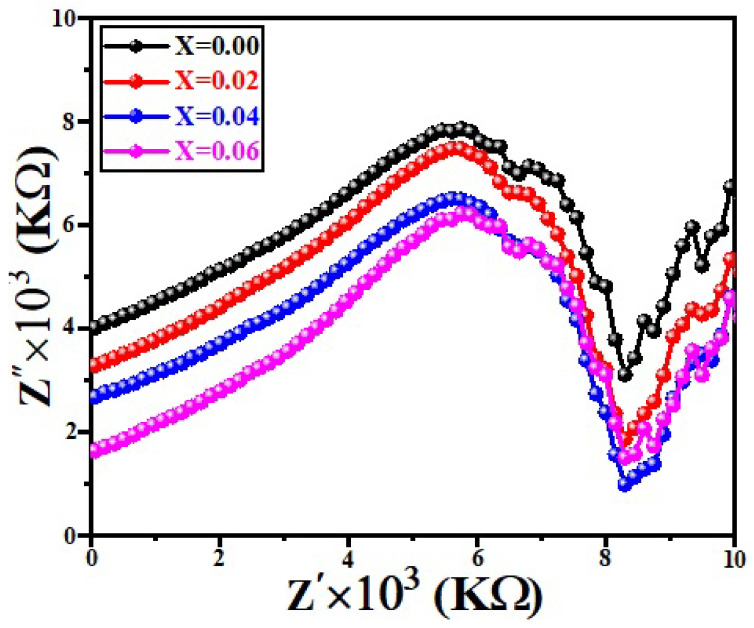
Cole-Cole Plots of (Sr_1−x_Ca_x_)_5_Ti_4_O_13_, 0 ≤ x ≤ 0.06 sintered ceramics.

The complex impedance spectroscopy mechanism is generally used to investigate the structural properties and bonding of the various types of materials, comprising the ferroelectric, ionic insulator, and linked ceramics under different experimental conditions [36]. The variation in real impedance Z′ and imaginary impedance Z″ of (Sr_1−x_Ca_x_)_5_Ti_4_O_13_, 0.0 ≤ x ≤ 0.06 sintered ceramics is shown in Figure 8. Initially, it was revealed that the magnitude of Z″ increases with Z′ and then decreases due to the release of space charge polarization [37]. It was observed that the magnitude of Z″ decreases by increasing the Z′ and Ca^2+^contents.

## 4. Conclusions

The solid solutions of (Sr_1−x_Ca_x_)_5_Ti_4_O_13_, 0.0 ≤ x ≤ 0.06 sintered ceramics was synthesized by conventional solid state method. The structural, microstructural, optical, and dielectric properties of all the samples have been investigated. The XRD patterns revealed the tetragonal phase with space group (I4/mmm). The SEM image revealed that the grain size and porosity increase with increasing Ca^2+^ contents, which was due to the difference of ionic radii. The results of UV spectroscopy declared that the bandgap energy increases from 1.68 eV to 2.24 eV along with increasing Ca^2+^ concentrations. The good values of dielectric properties (i.e., ε_r_ ~ 250, and tanδ = near to zero) in the frequency range from 100 Hz to 1 MHz was observed. It has been observed that the magnitude of Z″ increases with Z′ and Ca^2+^ contents. The overall findings are suitable for the application of dielectric devices.

## Figures and Tables

**Table 1:** Volume and Lattice parameters of Sr_n+1_Ti_n_O_3n+1_ (n = 4) sintered ceramics.

Contents	a = b (Å)	c (Å)	c/a	Error	Volume of Unit Cell (Å^3^)
0.00	3.8512	28.1253	7.3411	±0.8631	417.1471
0.02	3.8518	28.3951	7.3719	±0.8643	421.2800
0.04	3.8523	28.5505	7.4113	±0.8651	423.6955
0.06	3.8615	28.8975	7.4837	±0.8664	430.8958

## Data Availability

Generated data should be publicly available and cited in accordance with journal guidelines.
